# MiRPara: a SVM-based software tool for prediction of most probable microRNA coding regions in genome scale sequences

**DOI:** 10.1186/1471-2105-12-107

**Published:** 2011-04-19

**Authors:** Yonggan Wu, Bo Wei, Haizhou Liu, Tianxian Li, Simon Rayner

**Affiliations:** 1 Bioinformatics Group, State Key Laboratory of Virology, Wuhan Institute of Virology, Chinese Academy of Science, Wuhan, 430071, PR of China; 2 State Key Laboratory of Virology, Wuhan Institute of Virology, Chinese Academy of Sciences, Wuhan, Hubei, 430071, PR of China; 3 Department of Biological Sciences, Texas Tech University, Lubbock, TX, 79409-3131, USA

## Abstract

**Background:**

MicroRNAs are a family of ~22 nt small RNAs that can regulate gene expression at the post-transcriptional level. Identification of these molecules and their targets can aid understanding of regulatory processes. Recently, HTS has become a common identification method but there are two major limitations associated with the technique. Firstly, the method has low efficiency, with typically less than 1 in 10,000 sequences representing miRNA reads and secondly the method preferentially targets highly expressed miRNAs. If sequences are available, computational methods can provide a screening step to investigate the value of an HTS study and aid interpretation of results. However, current methods can only predict miRNAs for short fragments and have usually been trained against small datasets which don't always reflect the diversity of these molecules.

**Results:**

We have developed a software tool, miRPara, that predicts most probable mature miRNA coding regions from genome scale sequences in a species specific manner. We classified sequences from miRBase into animal, plant and overall categories and used a support vector machine to train three models based on an initial set of 77 parameters related to the physical properties of the pre-miRNA and its miRNAs. By applying parameter filtering we found a subset of ~25 parameters produced higher prediction ability compared to the full set. Our software achieves an accuracy of up to 80% against experimentally verified mature miRNAs, making it one of the most accurate methods available.

**Conclusions:**

miRPara is an effective tool for locating miRNAs coding regions in genome sequences and can be used as a screening step prior to HTS experiments. It is available at http://www.whiov.ac.cn/bioinformatics/mirpara

## Background

MicroRNAs are a family of ~22-nucleotide small RNAs that regulate gene expression at the post-transcriptional level [1]. MiRNAs play important roles in a number of key biological processes including developmental timing [2], tissue growth [3], apoptosis [3,4] and hematopoietic differentiation [4]. To date, 15172 miRNAs have been identified in 144 different species (according to miRBase 16.0 released in Sep 2010) [5,6]. Most of these miRNAs are considered to share a similar biogenesis mechanism: Firstly, transcribed RNAs form hairpin like structures (known as pri-miRNAs) that are incorporated into the Microprocessor and cleaved into pre-miRNAs [7,8]. The pre-miRNAs are then exported to the cytoplasm with the help of carrier proteins such as Exp-5 [9], where they are cleaved into small miRNA duplexes (~22-bp) by Dicer [10,11]. These small miRNA duplexes are unwound by Helicase into two independent strands--the passenger strand and the guide strand. The former is usually quickly digested while the latter is loaded into an Argonate (AGO)-containing protein complex known as RISC [12,13].

These mature miRNAs target multiple sites, producing translation repression and gene silencing; thus, identification of miRNAs and their targets can aid understanding of regulatory processes. Most miRNAs have been identified using techniques such as molecular cloning, Northern Blot or real-time PCR. More recently, high throughput sequencing has been adopted as a means of rapidly identifying larger numbers of miRNAs. However, a single run is still relatively expensive, detection efficiency is as low as 1 in 10,000 [14,15], and there is no guarantee that a run will identify any new sequences [16]. An additional limitation of all experimental methods is that they are inherently biased towards miRNAs that are highly or ubiquitously expressed and miRNAs expressed at low levels or in limited cell types may not be readily recovered[14].

The use of computational prediction tools can complement experimental studies by (i) identifying additional putative miRNAs that may not be detected by standard experimental methods and (ii) in the case of high throughput sequencing experiments, serving as a useful pre-sequencing step to determine the possible yield from such a study.

Many analytical methods for miRNA prediction already exist and employ a variety of approaches. Based on the approach used, these methods can be broadly classified into three categories.

In the first category, it is assumed there is some evolutionary constraint that conserves miRNAs across different species. In such methods, a sequence and/or secondary structure homology search is first applied between the primary sequences and known pri-miRNAs. Then several other parameters such as minimum free energy (MFE) or the number of base pairs are considered in order to further filter the candidates. Software using this approach include: miRAlign[17], RNAmicro[18], miRPred[19], miRseeker[20], MIRcheck[21], miRscan[22], PalGrade[23], miRFinder[24] miREval[25] and miRNAminer[26]. These methods are effective for identifying miRNAs from the sequence that are closely conserved in related species; for identification of miRNAs in more divergent sequences or miRNAs that are structurally distinct from previously identified sequences, these tools are less effective.

The second category attempts to identify key structural or compositional features of miRNA sequences that can be used to distinguish putative miRNAs from a broader range of candidate sequences. miRank[27] uses random walks to generate a pool of most probable miRNAs from a sequence, based on the properties of all possible sequence triplets and the mean free energy (MFE) of the predicted hairpin structure. This approach can correctly identify many miRNAs but also predicts many false positives. A similar situation occurs with BayesMiRNAfind, which uses a Bayes classifier to analyze properties of sequence composition and structure, primarily defining structural features in terms of the distribution of paired and unpaired bases in the hairpin structure[28]. In this case, the large numbers of false positives are removed by an additional filtering step to reduce the results to a more manageable size. The drawback is that the method is computationally intensive and analyzing sequences longer than a few hundred base pairs can take a prohibitively long time.

In the third category, prediction tools have been developed recently that try to consider the problem from a biological perspective. Both Microprocessor SVM and miRNA SVM attempt to identify important features of Drosha processing sites for miRNAs prediction. When used in combination with other miRNA prediction software, these tools can provide an additional validation layer for putative miRNAs and have achieved high sensitivity in an analysis of the human genome[29].

The limitation of all of these prediction tools is that they either are relatively species or miRNA family specific, or that they are computationally expensive and can only analyze small numbers of short sequences in a reasonable time. As more miRNAs have been identified, the number of homologous families and the diversity of the sequences has increased. This is supported by recent reports on the discovery of different miRNA maturation enzymes in different species, e.g, 4 different Dicer proteins have been reported in Arabidopsis[30] and two were reported in Droshophila[11], indicating that the miRNA use different pathways in different species[31].

With the advent of high throughput sequencing, there is a need for miRNA prediction software that can support such studies by analyzing large numbers of genome scale sequences in a reasonable time and which can be applied to a broad range of species without retraining. In this work, we first reviewed experimental studies that identified physical characteristics of the miRNA, pre-miRNA and pri-miRNA and which were found to influence the Biogenesis process and the generation of mature miRNA. Based on these characteristics we defined a number of parameters to describe a sequence and used them as input to an SVM. We trained two SVMs against experimentally verified plant and mammalian sequences from miRBase and a third SVM against all experimentally verified sequence data from miRBase data and evaluated these models against a range of positive and negative sequences to evaluate predictive abilities.

## Implementation

### Secondary Structure Prediction

Secondary structure prediction was performed using UNAFold [32,33]. ct2out (also written by the UNAFold authors) was used to transform the results from CT format to a text representation of the structure which was subsequently parsed out by our software. The formed hairpins were also subject to the restriction that the total stem length could not be longer than 60 nt to stop the formation of additional unstable pairing at the base of the stem.

### Parameter Selection

**Figure 1:**
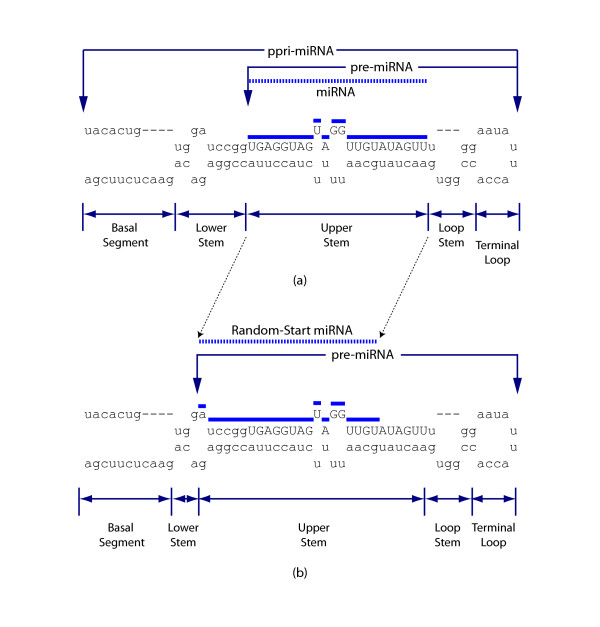
**Properties of pri-miRNA and negative test sequences**. (a) Key Structural Components of pri-miRNA. Following Han, J. et al [34] the secondary structure of a pri-miRNA sequence was assumed to be characterized by a combination of the following five features: Basal Segment, Lower Stem, Upper Stem, Top Stem and Terminal Loop. In this paper, the pre-miRNA was defined as the processed product of Drosha in the nucleus while the pri-miRNA was the combination of pre-miRNA and its flanking sequences. All five features were not present in all sequences, but every pri-miRNA contained a Terminal Loop and Upper Stem. (b) Construction of negative sequences used for SVM training. These sequences were based on experimentally verified pri-miRNA, but the start position of the miRNA was randomly shifted (by at least 5 bp) within the sequence. These negative sequences were called Random-Start miRNA.

The parameters used as input to the SVM were defined in terms of the pri-miRNA, pre-miRNA & miRNA sequences. The pre-miRNA was defined as the processed product of the Microprocessor complex in the nucleus while the pri-miRNA was the combination of pre-miRNA and its flanking sequences prior to processing. miRBase doesn't contain true pri-miRNA sequences but entries often have flanking sequences of variable length and so these were included in the analysis. Sequences containing these flanking regions are subsequently referred to as partial-pri-miRNAs or ppri-miRNAs for short. A ppri-miRNA sequence was considered to consist of five distinct components[34]: the Basal Segment, the Lower Stem, the Upper Stem, the Top Stem and the Terminal Loop (Figure 1), although the Upper Stem and the Terminal loop were the only features that were present in all ppri-miRNA sequences. We also defined the Internal Loop to be the largest bulge that occurred on either strand of each of the double stranded features and included additional parameters that were defined in terms of this feature.

**Figure 2:**
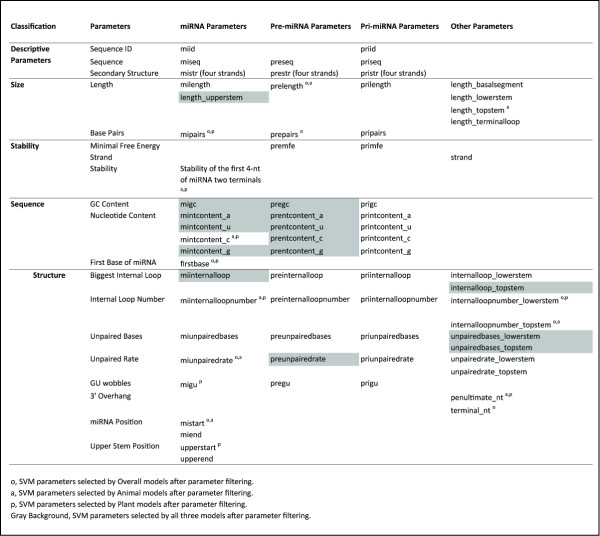
**Parameters defined for miRNA prediction**. A total of 77 parameters were initially selected that described properties of the pri-miRNA, pre-miRNA and miRNA sequences. These parameters were initially classified as Descriptive or related to the Size, Stability, Sequence or Structure properties. Parameters that were too broad in their range of values for use with an SVM were removed before training and parameter filtering was used to reduce the the remaining set to a subset of key parameters that were sufficient for accurate miRNA prediction. o - SVM parameters selected by the Overall models after parameter filtering, a - SVM parameters selected by the Animal model after parameter filtering, p - SVM parameters selected by Plant models after parameter filtering, Gray Background - SVM parameters selected by all three models after parameter filtering. See Materials and Methods for full details.

We first reviewed previous experimental studies that identified sequence features that influenced processing of pri-miRNA into mature miRNA (Additional File Table S1). Based on this, a set of 77 biological related parameters were defined that described various features of the miRNA, pre-miRNA, ppri-miRNA sequences and also for the five individual ppri-miRNA components. As a first step, these parameters were classified as one of five categories: Descriptive (ID, sequence and secondary structure string); Size (Length and number of paired bases in the hairpin structure), Stability (Mean Free Energy of secondary structure), Sequence (nucleotide content, GC content and first base of miRNA) and Structure (Internal Loop properties, number of unpaired bases, unpaired rate - the proportion of unpaired bases in a feature, GU wobble and the 3' overhang where the Lower Stem meets the miRNA start). These parameters are summarized in Figure 2.

### SVM Training Data

Positive Data: All 6330 experimentally verified miRNA sequences in miRBase release13.0 were screened for inclusion in the positive data set. In miRBase, the reported secondary structures were predicted by a variety of RNA folding software packages. Therefore, for consistency, all miRNA secondary structures analyzed in this study were recalculated using UNAFold. Structures with budding stems or with miRNA located in the terminal loop were excluded, leaving a total of 5576 sequences.

**Figure 3:**
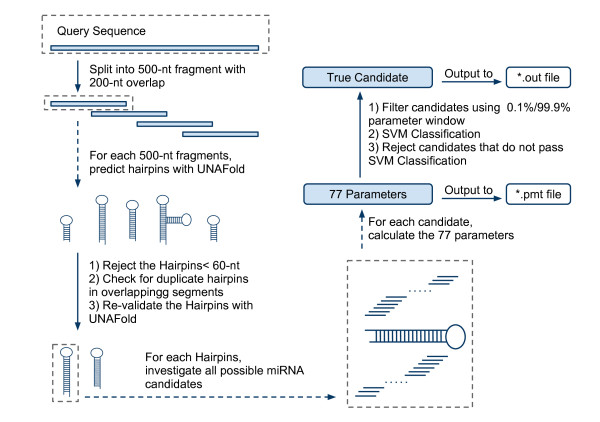
**miRPara prediction pipeline**. (i) if query sequence is longer than 500 nt it is split into overlapping fragments to prevent formation of long range structures. (ii) secondary structure of each fragment is predicted and if it forms a hairpin structure it is classified as a pre-miRNA candidate and passed to next step. (iii) all possible miRNA that can occur within the putative pre-miRNA are generated and, along with the parent pre-miRNA, are passed in turn to the SVM for classification. (iv) all parameters of the pre-miRNA/miRNA pair are calculated and passed through a filtering step. If the SVM classification is positive and the parameters pass the filtering step, the pair is classified as a true pre-miRNA/mi-RNA pair.

Based on the species category used in miRBase, the remaining 5576 experimentally verified pri-miRNA sequences were further separated into Metazoa, Viridiplantae and Viruses and used as positive input data for three corresponding SVMs (the number for a fourth group comprising Mycetozoa was too small to train an independent SVM); the miRNAs corresponding to these three groups were renamed as animal (4886 miRNAs), plant (1215 miRNAs) and virus (227 miRNAs) respectively. Another SVM model named overall was trained from all EV miRNAs and used for global predictions or for predictions on sequences not belonging to any of the three groups. A schematic of the miRNA prediction pipeline is shown in Figure 3.

Negative Data: The selection of a suitable negative dataset is important for a well trained SVM classifier. If the sequences are too artificial, e.g. completely random, then there is a risk that the SVM will not be well trained to distinguish between different categories of real biological sequences. Conversely, if the negative dataset is too similar to the positive dataset, the SVM will be unable to find a way to adequately distinguish between these two data. We investigated several different types of negative sequences and finally selected negative sequences with ppri-miRNAs that were identical to the positive sequences, but with modified mature miRNAs that had a start position that was located on the same strand as the real miRNA but which was randomly shifted by at least 5-bp from the true start position (Figure 1). These type of negative sequences were called Random-Start sequences.

Positive Data to Negative Data Ratio: Since the negative dataset had identical pri-miRNAs to the positive dataset, there was a risk of under or over-training the SVM. We therefore tried training the SVM with different ratios of positive and negative training data ranging from 1:1 to 1:20 for each group to examine the effect on performance. These different ratios are subsequently referred to as 'Levels' in this report. Each Level contains the same EV positive dataset but differs in the number of negative sequences that were included in the training data. So, Level 1 contained positive and negative sequences in the ratio 1:1, Level 2 in the ratio 1:2 and so on.

SVM Models: Three SVM prediction models, animal, plant and overall were trained based on the data listed above. We also attempted to train a model based on virus sequences, but there was insufficient data to do this. However, since all virus miRNAs in miRBase are associated with animal viruses, we also used the animal model to see whether it was effective at predicting virus miRNAs. To see the effect of sample number on SVM training, models with only 1000 randomly selected EV miRNAs and 1000 negative sequences were also trained but their accuracy was much lower than the four models trained with the full dataset (data not shown).

### Parameter Filtering

Parameter filtering was applied independently for the animal and plant groups. Of the 77 parameters in the initial set, 21 were removed after being flagged as unsuitable for SVM input. An additional 14 were removed prior to filtering because the sequences in the negative dataset were based on the positive sequences and were indistinguishable between the datasets. This left a set {P} of 42 parameters (Figure 2). We then used a greedy algorithm to investigate whether there was a subset of {P} that was capable of discriminating between negative sequences and true miRNAs without significant loss of specificity or sensitivity, First of all the remaining 42 parameters were used in turn to independently train the SVM using three sets of 500 EV sequences as positive datasets, and three sets of 500 Random-Start sequences as negative datasets. The 10 highest scoring (i.e. most accurate) parameters, averaged over the three datasets, were then retained as a set {S} of seed parameters. Every element si of S was then used as a seed for a parameter chain of length two (si pj; where si ≠ pi) and each parameter was investigated in turn. The 10 highest scoring pairs were then retained as a set of seed chains for the next round to identify the highest scoring parameter triplets. This was repeated until the 10 highest scoring chains for all 42 parameter chains had been estimated and no further parameters were remaining. A plot of chain length versus accuracy was then generated to identify the highest scoring chain length. It was found that for each 42 parameter chain, the highest score was obtained for the parent chain of length 25. Furthermore, the first 25 parameters were identical in each chain for each model and so they were selected as the filtered subset.

### Range Filtering

To investigate the effect of windowing parameters prior to SVM training we generated a distribution for each parameter for the EV sequences. Using SPSS http://www.spss.com/ we tested windows with 5%/95%, 1%/99% and 0.1%/99.9% limits and any sequence with values falling outside these limits was rejected. We finally selected 0.1%/99.9%, which passed 97.2% of the data. The sole exception was the MFE of the pri-miRNA structure for which we applied the constraint that MFE <-20 kcal/mol (the value of 5%/95%). This reduced the overall percentage to 95.2% but improved the accuracy of prediction as it removed many relatively unstable structures associated with ppri-miRNAs in the negative dataset.

### Support Vector Machine (SVM)

The SVM was implemented using the LIBSVM library[35] with a perl binding module http://search.cpan.org/~lairdm/Algorithm-SVM-0.13/lib/Algorithm/SVM.pm.

### SVM Training

The SVM was trained against the data described above using 2×, 50× & 100× cross-validation with default values for γ and C. Datasets were then optimized for γ and C using the grid selection approach recommended by the LIBSVM authors[36].

SVM performance was measured using Accuracy, Sensitivity and Specificity according to the following definitions

Where TP = number of predicted true positives, TN = number of predicted true negatives, FN = number of predicted false negatives and FP = number of predicted false positives. Some of the miRPara predictions correctly matched miRBase entries, but were lacking one or more nucleotides from either end of the sequence. Since the goal of the software is to identify potential miRNA sequences for further study, predicted sequences with up to three nucleotides missing from either end of the true sequence were considered positive matches.

### Test Data

#### MiRPara Short Sequence (100-nt) Test Data

To investigate the performance of the three models in an ideal situation, a series of 100 randomly selected EV sequences were used as the positive datasets. A length of 100 nt was chosen since this length is sufficient to capture most real pre-miRNAs. The performance was tested independently for all three models, and for each model three different sequence sets were tested. For the negative controls, 100nt fragments were randomly selected from three different nucleotide sequences which were downloaded (three for each model) from the NCBI website http://www.ncbi.nlm.nih.gov/. These random sequences were then passed to UNAFold for secondary structure prediction and any sequences that formed a hairpin structure were added to the negative dataset.

The accession numbers were as follows:

animal: Homo sapiens (NG_008663, NM_000129 and NM_001731);

plant: Arabidopsis thaliana (NM_119826, NM_122126 and NM_203075);

virus: Epstein Barr virus (V01555), Marek's disease virus (AF243438) and Rhesus lymphocryptovirus (AY037858);

overall: Homo sapiens (NG_008663), Arabidopsis thaliana (NM_119826) and Epstein Barr virus (V01555).

#### MiRPara Long Sequence (~10000-nt) Test Data

To test the ability of miRPara to extract miRNAs from long sequences, three different groups of 100 positive and negative data sets were used. Each positive sequence consisted of the original ppri-miRNA sequence from the miRBase entry with a 5000-nt flanking sequence on each side. To find the flanking sequence, a perl script was used to BLAST each ppri-miRNA against the NCBI database to identify the parent sequence; the long sequence with the 5000-nt flanking regions was then extracted from this parent. The ROC curve negative data was created in the same way as the negative dataset for the 100nt sequence test.

#### Accuracy versus Sequence Length

To investigate whether there was any correlation between sequence length and accuracy a series of sequence datasets were generated ranging in length from 100 to 2000 in 50nt increments. These sequences were identical to the MiRPara Long Sequence (~10000-nt) Dataset but with different size flanking sequences to generate the required sequence size. miRPara was run against each set and the accuracy was calculated.

#### Virus Genome Sequences

To test the prediction ability against genome sequences, the three full length virus sequences used in the short sequence test were submitted directly to miRPara and prediction results compared to experimentally verified entries in miRBase. Genome lengths were: Epstein Barr virus (172281 bp), Marek's disease virus (177874 bp) and Rhesus lymphocryptovirus (171096 bp )

Other RNA Testing Data: To see whether the trained model could distinguish between miRNA and other types of non-coding RNAs, miRPara was tested against rRNA and tRNA sequences. Human rRNA sequences were downloaded from the NCBI website with the following accession numbers: 18s rRNA(X03205), 28s rRNA(NR_003287), 5.8s rRNA(NR_003285), 5s rRNA(V00589). 631 human tRNAs were downloaded from the genome tRNA database http://gtrnadb.ucsc.edu/[37].

#### Data for Comparison with Other miRNA Prediction Software

To compare miRPara with other miRNA prediction software, three types of 100 test sequences were used:

1) known miRNAs. Here 'known' refers to miRNAs that were identified before the earliest miRNA prediction software that was used in this study and would therefore have been available as training data to all the programs. This group was therefore constructed from EV miRNAs available in miRBase7.1 (Released on Oct. 2005) and which were also present in miRBase13.0;

2) new miRNAs. This consisted of 1344 EV miRNAs extracted from miRBase14.0 which were not present in miRBase13.0, these miRNAs were tagged as "NEW" in this release;

3) negative sequences. These 100-nt fragments were randomly extracted from the same sequences as described above in "miRPara Short Sequence Test Data"; equal numbers of miRNAs were generated from animal, plant and virus sequences respectively.

All test sequences were randomly selected.

A complete list of identified miRNA/pri-miRNA prediction software is given in Additional file Table S2. As the goal of this study was to identify miRNA, packages that only predicted pre-miRNA were not considered. Of the five packages we found, miRFinder[24] proved to be too slow to analyze the large test data set and miRank[27] couldn't be tested as it requires the commercial software package MATLAB with the bioinformatics toolbox. The three remaining packages were miRAlign[17], BayesMiRNAfind[27] and mirEval[25]. BayesMiRNAfind was further selected to test the miRNA prediction performance among different species because its prediction algorithm does not rely on sequence homology searches. The ppri-miRNA for the randomly selected test sequences were submitted to each program without modification. In this test, miRPara was used to report exact mature miRNAs rather than most probable coding region, i.e., the test sequence was only considered a true positive if the predicted start and stop position exactly matched the start and stop position of the miRBase entry.

Benchmarks: To examine the performance of miRPara with sequence length, a series of test sets containing different length sequences were created. Each set contained 10 sequences randomly selected from miRBase and data sets were created for 100, 200, 500, 1000, 2000, 5000 & 10000nt sequences. The different lengths were created in the same way as the Long Sequence (~10000-nt) Test Data but using different size flanking regions. The run times were recorded on a MacBookPro with dual core P8700 CPUs @ 2.53 GHz and 4 GB memory running Ubuntu release 10.10.

### Perl scripts

A combination of Perl Scripts were used for the sequence preparation, SVM training and miRNA predictions with the trained model. The complete scripts are available from our website http://www.whiov.ac.cn/bioinformatics/mirpara.

## Results

### I-miRPara Construction

#### Parameter Filtering

**Figure 4:**
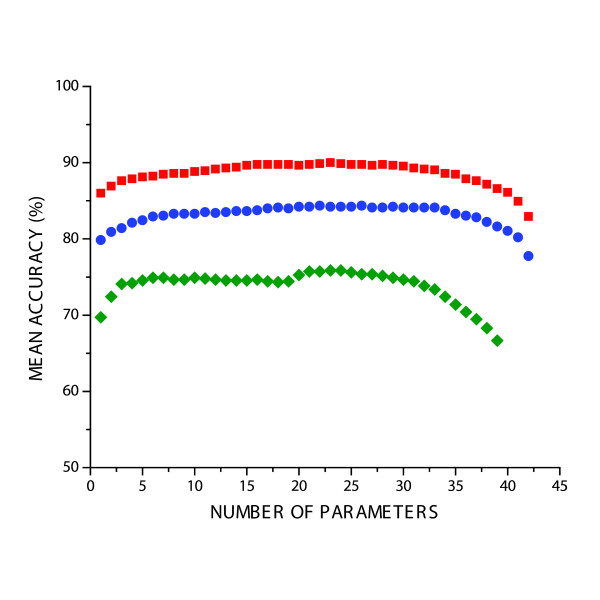
**Parameter Filtering Curve**. Variation of accuracy with number of parameters used in SVM training for the *Overall*, *Animal *and *Plant *models. To investigate whether a smaller set of parameters were sufficient for training, the SVM was successively retrained for a growing set of parameters that was incremented by one for each training cycle. Beyond 25 parameters, there was little improvement in accuracy, and when more than 30 parameters were used, the accuracy began to drop with significantly less accuracy achieved when all 44 parameters were used (far right of plot). See Materials and Methods for full details. Arrows indicate the number of parameters selected for each model.

To investigate the effect of number of parameters, parameter filtering was used to see whether a subset of key parameters existed that could retain the accuracy of the full set of 42 parameters (Materials and Methods). Parameter selection was performed separately for the overall, animal and plant models and a key set of 25, 24 and 24 parameters for the overall, animal and plant models respectively were identified that provided a significant improvement in the accuracy of the trained SVMs compared to the full set of parameters (Figure 4).

A similar set of key parameters was selected for the three different models although these parameters covered all categories (Size, Stability, Sequence and Structure)[31]. However, most of the parameters selected in the different models overlap with at least one other model, and half of the parameters are shared amongst all three models (Figure 2). In particular, nucleotide content and GC content in both miRNA and pre-miRNA were selected for all three models.

#### SVM Training and Testing

Using the selected parameters, three different SVM models, overall, animal and plant, were trained as described in the Materials and Methods. The animal model was also used to classify the virus sequences. These models were then tested with positive (experimentally verified miRNAs) and negative (Random-Start sequences) data (See Materials and Methods).

**Figure 5:**
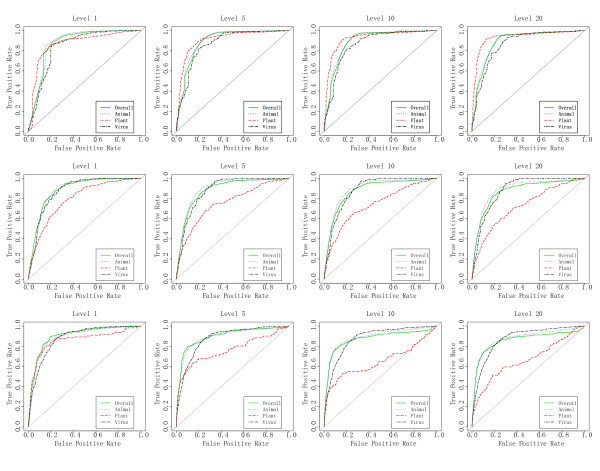
**ROC curves for training and test data**. ROC curves for training and test data at different ratios of positive to negative data. Level 1 corresponds to a 1:1 ratio of Positive to Negative data, whereas Level 20 refers to a 1:20 ratio of positive to negative data. From left to right curves are shown for Level 1, Level 5, Level 10 & Level 20. *Top row*: ROC curves for training sets *Overall *(green), *Animal *(blue), *Plant *(red) &*Virus *(black). *Middle Row*: ROC curves for 100nt test datasets. Negative datasets comprises sequences that are predicted to form a hairpin loops but which are not in miRBase. *Bottom Row*: ROC curves for 10000nt test dataset. Positive dataset contains known pre-miRNAs from miRBase which have 5000nt flanking sequences identified by BLASTing against the NCBI nt database. Negative datasets comprises sequences that are predicted to form a hairpin loops but which are not in miRBase and flanking sequences were identified in the same manner.

Cross validation results are shown in Table 1 and ROCs curves for the models trained at different levels are shown in Figure 5 (top row). As the level was increased from 1 to 20, there was a slight increase in predictive ability which was likely a consequence of the similarity between the positive and negative datasets.

**Table 1:** Cross validation results

Cross Level	2X	10X	20X	30X	40X	50X	60X	70X	80X	90X	100X
Overall	76.89	77.58	77.50	77.61	77.67	77.51	77.67	77.62	77.62	77.66	77.50
Animal	80.06	80.20	80.37	80.30	80.43	80.42	80.35	80.29	80.40	80.38	80.43
Plant	68.44	71.29	72.46	71.04	71.60	72.15	71.97	72.09	72.22	71.84	72.09

Cross validation results at different levels for the Overall, Animal & Plant models

All three models demonstrated good prediction capability. Surprisingly, the animal model applied to the virus data also showed good performance; this might be because, compared to the other datasets, the virus dataset contains a relatively small set of sequences from a narrow range of viruses and contains less variation.

### II-Testing miRPara against small fragments

We first examined how well the trained models performed against a series of ~100-nt fragments, which are of sufficient length to contain a pre-miRNA sequence and represent an ideal scenario. For each of the animal, plant, virus and overall sequence sets, three groups of negative sequences comprising 100-nt fragments were generated, subject to the requirement they each form a hairpin structure (Materials and Methods). At the same time, three groups of 100 randomly selected EV sequences were used as positive controls and predictions were generated for all twenty Levels.

The results are shown in ROC plots. (Figure 5 middle row). In each case, slightly better predictions are obtained when equal numbers of positive and negative data are used. Thus, for the following sections, Level 1 was used for predictions unless stated otherwise. Overall and Animal return the best predictions with slightly worse results for plant and virus.

The plant model showed marginally worst performance. The reason for this difference isn't clear, but it may be a consequence of the plant dataset containing miRNAs from a wider range of species (and hence greater diversity) as well as the presence of additional small RNAs that only exist in plants. For example, trans-acting siRNA (ta-siRNA) has only been reported in plant[38] and it needs miRNA to trigger the production of siRNA, which means the ta-siRNA and miRNA share parts of the same processing pathway.

### III-Testing miRPara Against Other Types of Non-coding RNAs

We next considered whether miRPara could distinguish between miRNA and other types of non-coding RNA molecules by considering test datasets of human rRNA and tRNA sequences. As many of these sequences also contain structural and sequence composition features that are hairpin like and, given the different functions of these molecules, it is important to test the ability of software to differentiate these types of RNA

#### rRNA

rRNAs are components of the ribosome and play an important role in protein synthesis. Although the structure and the function of rRNA are widely different from miRNA, 5s, 5.8s, 18s and 28s rRNA have average lengths of 121-nt, 156-nt, 1869-nt and 5035-nt respectively, all of which are long enough to contain pri-miRNA like structures. Candidate human rRNAs were downloaded from the NCBI website as described in the Materials and Methods. miRPara did not predict any miRNAs in the 5s, 5.8s and 28s rRNAs but for the 1869-nt 18s rRNA sequence, 3 pre-miRNAs and 5 miRNAs were predicted. However, two of these predictions matched entries in miRBase and the third showed high homology to another entry. This is consistent with the results from an earlier experimental study to identify miRNA identification that used rRNA as the control and which also cloned two positive sequences from this negative set[39].

#### tRNA

tRNA transfers amino acids to growing polypeptide chains at the ribosomal site of protein synthesis during the translation process. It possesses primary, secondary and tertiary structure and so could conceivably be misidentified as miRNA by miRNA prediction software. To test this, 631 tRNA candidates were downloaded from the UCSC tRNA database and miRPara was used to predict miRNAs at Levels 1 to 20. 54 and 33 pri-miRNAs were identified by miRPara at Level 1 and Level 20 respectively. Several of these false positives contained regions that formed highly stable hairpins and which also contained perfect matches to almost complete segments of mature miRNA entries in miRBase.

### IV-Testing miRPara prediction against long fragments

The ultimate goal of our prediction software is to identify true miRNAs from long genome fragments. miRPara achieves this by cutting a long query sequence into a series of 500-nt fragments with a 200-nt overlap and analyzing each fragment in turn (see miRPara pipeline, Figure 3). These values were chosen because most pre-miRNA fragments are less than 200nt and stable ppri-miRNA structures are generally formed from nucleotides located in a single region, rather than from interactions between two distant sequences. The program was tested against three groups of 100 positive sequences, each containing one true EV pri-miRNA, with 5000nt flanking sequence on either side (Materials and Methods).

The results are shown as ROC plots (Figure 5 bottom row) and are noticeably better than the results for the short sequences. The difference arises because the flanking sequences contain additional positive and negative candidates that are classified by the software. For all three models and all four test sets, the software gave consistently good prediction results.

**Figure 6:**
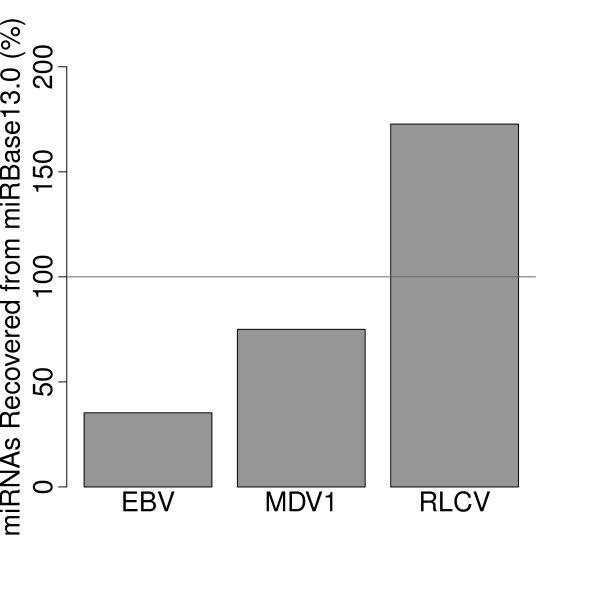
**miRPara prediction accuracy for three full length virus genomes**. miRPara prediction results for three full length virus genomes: Epstein Barr virus (ebv), Marek's disease virus (mdv1) and Rhesus lymphocryptovirus (rlcv). For Rhesus lymphocryptovirus, 19 additional miRNAs were predicted that were subsequently confirmed in the following releases of miRBase.

We also tested the software against three virus genomes. Full length sequences for Epstein Barr virus (ebv), Marek's disease virus (mdv1) and Rhesus lymphocryptovirus (rlcv) were submitted to miRPara and prediction results compared to miRBase entries for these viruses. Results are shown in Figure 6. Notably, for Rhesus lymphocryptovirus, 19 additional miRNAs were predicted that were subsequently confirmed in the following release of miRBase.

### V - Comparison with other miRNA prediction software

We next compared our software to other miRNA prediction software using a set of test data that examined their ability to (1) correctly predict true known miRNA, (2) make predictions for new miRNAs and (3) reject false miRNA. A list of all identified miRNA prediction software identified in the literature is given in Additional file Table S2. Since the goal of our software was to predict miRNA, we did not consider packages that only predicted pre-miRNA. Of these, three programs - miRAlign[17] and mirEval[25] based on homology searches) and BayesMiRNAfind[28] (based on a Naïve Bayes classifier) were finally selected. (See Materials and Methods for details about the test data and how the packages were chosen).

**Figure 7:**
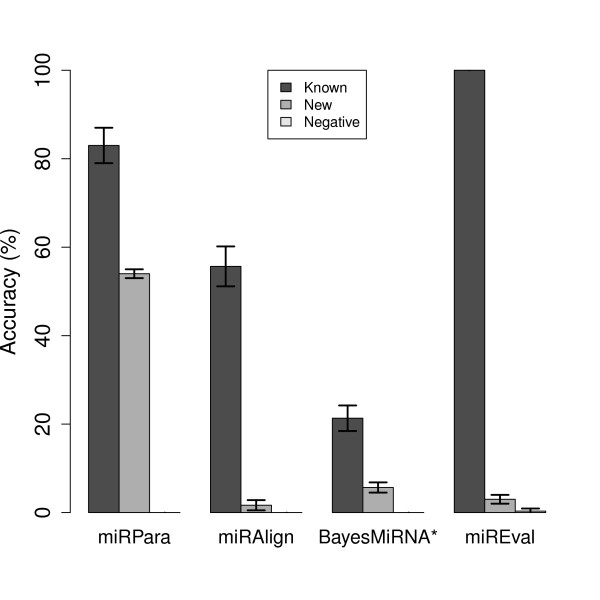
**Comparison of prediction abilities of miRPara and other miRNA prediction software**. The predictive ability of MiRPara was compared with four other current miRNA prediction software packages (BayesMiRNAFind, MiRAlign, MiREval and MiRPara). Software was tested with three data sets to test (i) the ability to correctly predict experimentally verified known miRNA (Known) (ii) make predictions for new miRNAs from a set of sequences that were new submissions in version 14.0 of miRBase and which had not been previously presented to any of the software packages (New) (iii) reject negative sequences containing no miRNA (Negative) Full details of how the datasets were generated are given in Materials and Methods. miRPara outperformed all other packages in all three categories with the exception of the positive prediction rate of miREval, but this package automatically searches miRBase for homology to every test sequence and thus matches every sequence. When presented with the new positive sequences, the performance is significantly worse.

The prediction results are summarized in Figure 7. All three packages could reject negative sequences but only miRPara and miREval appeared able to classify true positives effectively. Although miREval correctly predicted 100% of the sequences, one of its prediction strategies is to check a test sequence with sequences in miRBase. MiRPara predicted around 80% of the known miRNAs which was significantly better than the ~60% and ~20% that were predicted by miRAlign and BayesMiRNAfind respectively. However, the performance of these two packages in these tests is below what had been reported by the authors in their original publications. Nevertheless, the test sequences were randomly selected, rather than in a way which would yield some advantage to our software. One possible explanation is that these programs were trained against limited subsets of the full miRBase release; BayesMiRNAfind was trained against a broader range of mammalian sequences and returned the best results of the three. It therefore seems likely that the remaining two software packages would return improved results when tested against more specific mammalian datasets.

MiRPara also proved to be the most effective in predicting new miRNAs. The sequences in the "New miRNAs" dataset were experimentally validated new submissions in miRBase14.0, i.e., none of these new miRNAs were used during the training of miRPara (which was based on miRBase13.0). miRPara predicted ~50% of the new miRNA entries in miRBase14.0. The corresponding percentage for the homologous searching based packages was very low, around 1% for miRAlign and 3% for miREval. The performance of BayesMiRNAfind was slightly better (~5%) but still very low compared to miRPara. A complete list of new predictions that were not present in miRBase13.0 but which were verified in subsequent releases are given in Additional file Table S3.

**Figure 8:**
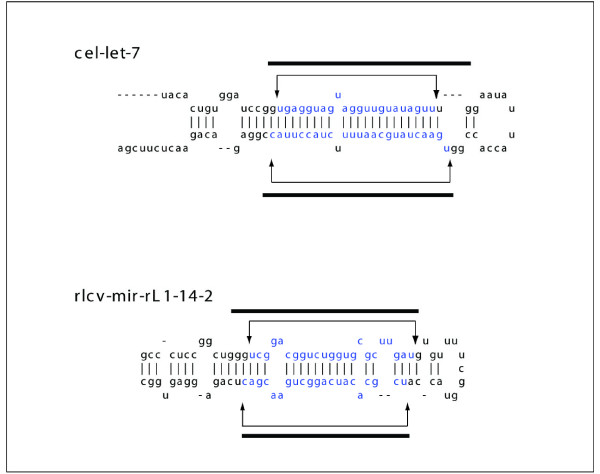
**Examples of miRBase predictions**. Figure shows two predictions for (a) cel-let-7, one of the first miRNA to be identified and (b) rlcv-mir--rl1-14-2, a miRNA predicted for Rhesus lymphocrpptovirus that wasn't in release 14 of miRBase, but which was subsequently verified in release 15.0 of the database. the thin line with arrows marks the exact location of the miRNA. the thick line marks the region that miRPara predicted to contain an miRNA sequence.

Two predictions are shown in Figure 8 along with some of the calculated parameters (Table 2). Figure 8a shows the prediction for cel-let-7, Figure 8b shows the prediction for rlcv-mir-rL1-14-2 in Rhesus lymphocryptovirus that was subsequently verified in release 14 of miRBase.

**Table 2:** predicted parameters for miRNAs 'rlcv-mir-rL1-14-2' and 'cel-let-7'

	rlcv-mir-rL1-14-2				cel-let-7			
								
	STRAND				STRAND			
Parameter	LOWER		UPPER		LOWER		UPPER	
	min	max	min	max	min	max	min	max
**Length Upper Strand**	20	24	20	26	20	27	20	24
**pre GC content**	0.517	0.536	0.517	0.547	0.340	0.386	0.365	0.403
**miRNA A content**	0.044	0.100	0.044	0.333	0.182	0.300	0.238	0.318
**miRNA C content**	0.174	0.250	0.174	0.364	0.000	0.000	0.208	0.292
**miRNA G content**	0.391	0.500	0.191	0.500	0.273	0.417	0.042	0.167
**miRNA U content**	0.200	0.304	0.100	0.304	0.350	0.455	0.350	0.450
**Pre-miRNA A content**	0.177	0.190	0.172	0.190	0.262	0.289	0.254	0.271
**Pre-miRNA C content**	0.222	0.233	0.222	0.235	0.128	0.158	0.143	0.164
**Pre-miRNA G content**	0.293	0.307	0.293	0.313	0.208	0.233	0.220	0.239
**pre-miRNA U content**	0.283	0.296	0.279	0.296	0.349	0.383	0.343	0.365
**miRNA largest Internal Loop**	2	2	2	2	2	4	2	3
**TopStem largest Internal Loop**	1	2	1	2	-1	3	-1	3
**miRNA No of Internal Loops**	2	4	2	4	2	4	2	3
**Lower Stem No of Unpaired Bases**	4	4	4	4	4	6	4	4
**Upper Stem No of Unpaired Bases**	2	5	2	5	0	3	0	3
**miRNA Unpaired Rate**	0.150	0.211	0.143	0.211	0.130	0.378	0.125	0.200
**Pre-miRNA Unpaired Rate**	0.164	0.192	0.148	0.192	0.208	0.233	0.220	0.239

Calculated parameters for the predictions shown in figure 5. Only parameters that were selected by parameter filtering are shown.

### VI - Benchmarks

**Figure 9:**
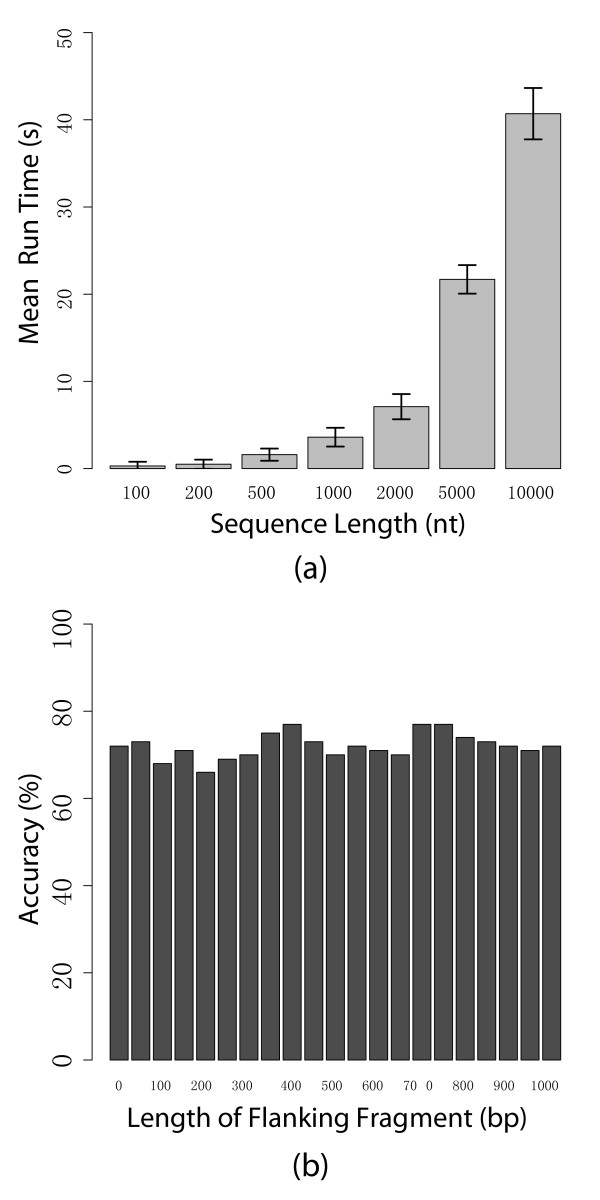
**Benchmarks for different datasets**. (a) Benchmarks for run time versus sequence length for sequences ranging from 100nt to 10000nt. A 10000nt sequence can be analyzed in less than a minute; the non-linear increase in run time as a function of sequence length is due to the number of secondary structures that are predicted (b) Accuracy versus sequence length. To ensure there was no loss of accuracy with longer sequences we also calculated the accuracy for the dataset to ensure there was no size effect.

miRPara is designed for analysis of genome scale sequences. We therefore examined the performance by analyzing a series of sequence sets ranging in length from 100 to 10000. We also examined the accuracy for each dataset to ensure there was no size effect beyond what was observed with the 100nt sequence set and longer sequences. Figure 9a shows the benchmark results. The non-linear increase in run time as a function of sequence length is due to the number of secondary structures that are predicted. Nevertheless, the program can analyze a 10000nt sequence in less than a minute with an off-the-shelf laptop and full length genome sequences can be analyzed in a few hours. One concern was whether accuracy might tail off as the sequence length was increased due to the formation of more complex secondary structures. Figure 9b shows a plot of accuracy against sequence length for sequences up to 10000nt and there is no evidence of dependency on sequence size.

## Discussion and conclusions

We have developed a new software tool, miRPara, for the prediction of miRNA and pre-miRNA sequences from DNA or RNA sequences of any length. There are three major differences between our approach and previously reported methods. First, rather than defining a broad range of parameters to describe the sequences, we examined results from experimental studies to determine parameters that appeared to be most relevant to the miRNA maturation process. These parameters were then used as inputs to a support vector machine (SVM). Second, instead of training a single model for all sequences, we trained three different SVM models, overall, animal & plant. (Attempts to train a fourth model for virus sequences was unsuccessful because of insufficient numbers of experimentally verified miRNAs). Finally, training separate models allowed us to use a significant portion of the data in miRBase, rather than restricting ourselves to subsets of the data. All three models showed good performance with high specificity and sensitivity and the animal model was also effective at predicting virus sequences.

A parameter filtering step was included to examine whether accuracy could be used by using a subset of parameters. Interestingly, step identified features that had previously been considered irrelevant in the characterization of pri-miRNA, pre-miRNA and miRNA. There has been much debate as to whether Nucleotide Content[40-42] and GC Content[43] are critical for miRNA processing. However, in this work, we found that nucleotide content for all 4 bases as well as GC content were key parameters for identification of both pre-miRNA and miRNA sequences (Figure 2). This is possibly because the SVM is combining these parameters in the training process, rather than analyzing them independently, which was the approach used in earlier studies.

Additionally, our results indicate that the size of miRNA secondary structure, length_upperStem, is more important than the length of miRNA----miLength. This result seems reasonable because Dicer, the enzyme that cleaves pre-miRNA into an miRNA duplex and thus determines the length of miRNA, only interacts with dsRNA, i.e., if there are some features that are involved in the determination of cleavage length, they are more likely to be related to the secondary structure of the miRNA.

Another interesting result was the selection of parameters describing properties of the Upper and Lower Stem in the pri-miRNA. Although this isn't consistent with the observation that the length of these features vary greatly amongst different pri-miRNA sequences, with several sequences failing to incorporate these features, the majority of the selected parameters are related to the unpaired bases in the region, i.e., these unpaired bases might contribute to some relevant structural feature that is important for slicer complex recognition or processing. However, this is purely speculative and further study is necessary and beyond the scope of this report.

Comparisons with other currently available miRNA prediction software (miAlign, miREval and miRNABayesFind) found miRPara was the most effective at identifying a broad range of known miRNAs and outperformed other packages in identification of new miRNAs. However, there is no information available about what training sets were used on the most current versions of these other software tools and it is likely they will give better performance when identifying miRNAs in specific mammalian sequences or retrained with different datasets. For more general analysis of genome sequences however, our software appears to be the most accurate and can analyze full length genome sequences with no upper limit on size.

The goal of developing the software was to complement HTS experiments and miRPara is primarily designed for use in wet-lab studies to screen long sequences for putative miRNAs as well as pre-testing miRNAs of interest to reduce bench search range. Because the miRNA maturation process is still not fully understood, it is not possible to identify the miRNA within the parent pri-miRNA with 100% certainty. This is a problem that exists with all miRNA prediction software. For example, miRNABayesFind provides the user with large numbers of candidate miRNAs, many of which are overlapping. Nevertheless, each individual prediction, even when overlapping, is considered a mature miRNA prediction. On the other hand programs such as miAlign, which assume the presence of some degree of sequence homology, are able to precisely identify miRNA candidates, but at the expense of rejecting many true miRNAs. Thus, rather than reporting individual mature miRNA predictions and their specific cleavage sites, we consolidate overlapping miRNA predictions into a single predicted miRNA coding region. Additional file Figure S1 shows an example where miRBase prediction results are combined with HTS data [44]; multiple miRBase predictions are clustered around multiple sequencing reads to define a region or miRNA 'hotspot' within the pri-miRNA that is generally no more than a few nucleotides wider than the real miRNA. These results are more straighforward to interpret, particularly for very long sequences and identifying these most probable miRNA coding regions allows smaller primer pairs sets to be used for sequencing cloned fragments and identifying the true miRNA fragments.

## Availability and Requirements

miRPara is an effective tool for locating miRNAs coding regions in genome sequences in a species specific manner. The software uses an SVM approach to train against known animal, plant and virus miRNA sequences in miRBase and can be easily retrained against more specific datasets or new releases of miRBase as they become available. The software can be run on a standard desktop computer and analyze full length genome sequences in a matter of hours. miRPara is written in Perl and can be run as a standalone application or as a remote service on a webserver. Both packages and source code can be accessed via our website http://www.whiov.ac.cn/bioinformatics/mirpara/. Although miRPara can parse any length sequences, we have set an upper limit 500-nt for the online version. For longer sequences users can run the standalone application.

## Abbreviations

miRNAs: microRNAs; SVM: Support Vector Machine; AGO: Argonate; RISC: RNA Induced Silencing Complex; MFE: Minimum Free Energy; ta-siRNAs: Trans-acting small interference RNAs; ppri-miRNAs: Partial Primary microRNAs; EV: Experimentally Verified; HTS: High Throughput Sequencing; ebv: Epstein Barr virus; Mdv1: Marek's disease virus; Rlcv: Rhesus lymphocryptovirus

## Authors' contributions

YW wrote the miRPara perl scripts, trained the SVM models and did the data testing and analysis, built the miRPara online websites and wrote the manuscript. BW wrote a java version of the software which will be released shortly. HL participated in miRPara website building and implemented the remote service on the server. SR organized the data set, performed data analysis, wrote additional data analysis software and wrote the manuscript. All authors read and approved the final manuscript

## Supplementary Material

Additional file 1 **Supplemental material**. Click here for file
