# Supplementary material for: MiRPara: a SVM-based software tool for prediction of most probable microRNA coding regions in genome scale sequences
Source: BMC Bioinformatics. 2011 Apr 19;12:107. doi: 10.1186/1471-2105-12-107 (PMC3110143; doi:10.1186/1471-2105-12-107)
Supplement: Additional file 1 — Supplemental material. [file 1471-2105-12-107-S1.DOC]

**Supplementary Figure 1.**

##
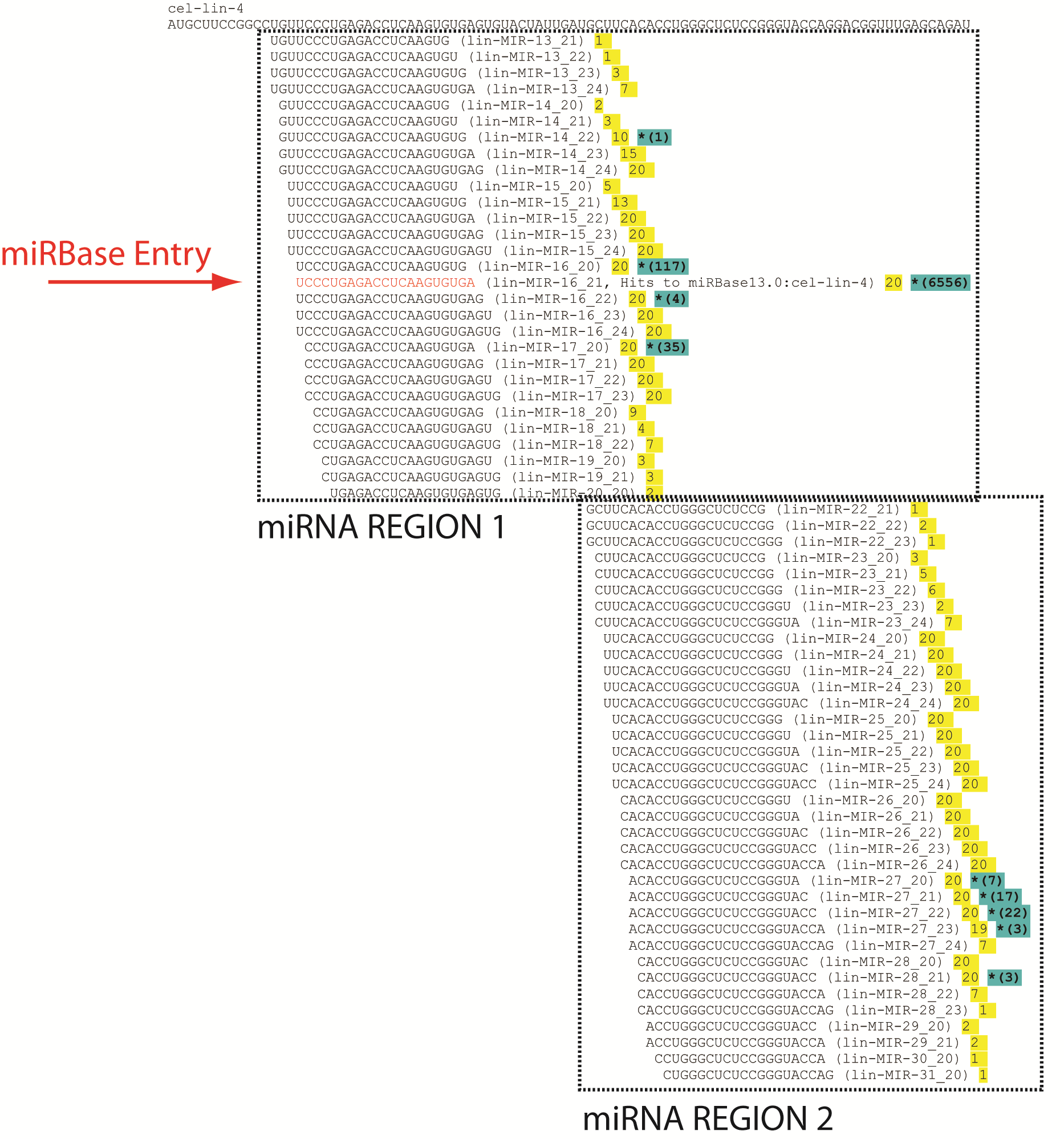


## Supplementary Figure 1. Sample miRPara prediction result combined with HTS data for post sequencing analysis.

Example of miRNAs predicted around a previously identified miRNA that was verified by experiment. All predicted miRNA candidates are shown. Name of each candidate is given in parenthesis. First number highlighted in yellow refers to level that was used to predict the candidate. Asterisk indicates miRNA detected by deep sequencing by Ruby et al [44]; second number in parentheses (highlighted in blue) indicates number of recorded reads in the study. boxes indicate reported miRNA Regions for each miRNA sequence set.

## Supplementary Table 1: Experimental studies investigating the properties of pri-miRNA and miRNAs

| Parameter | Parameter Value | Evidence |
| --- | --- | --- |
| PriLength | 60-to 80-nt | Summarized [1] |
|  | ≧76-nt | Experimental [2] |
|  | ~65-nt | Experimental [3] |
|  | >~70-nt | Experimental [4] |
|  | 90.4522±0.4164 (vertebrate) | Summarized [5] |
|  | 137.9175±2.0309 (plants) | Summarized [5] |
| Length of dsRNA | 100- to 450-nt (degradation increased.) | Experimental [6] |
|  | 59-nt | Experimental [6] |
|  | >150-bp | Experimental [7] |
|  | 49-bp (ineffective) | Experimental [7] |
|  | 78 mer | Experimental [8] |
|  | 300-nt | Experimental [9] |
|  | 540- and 400-nt (quite effective) | Experimental [9] |
|  | 200- and 300-nt (less potent) | Experimental [9] |
|  | 50- or 100-nt (inert) | Experimental [9] |
|  | 38- to 501-bp | Experimental [10] |
|  | 29- to 36-bp (not effective) | Experimental [10] |
|  | 30-, 40-, 50-, 70- and 130-bp | Experimental [11] |
|  | ≧27-bp | Experimental [12] |
|  | 40- to 45-bp (inactive) | Experimental [12] |
|  | 21-bp (inactive) | Experimental [12] |
|  | 22-bp (low efficiently) | Experimental [13] |
| PreLength | 70- to 80-nt | Summarized [11] |
|  | 60- to 90-nt | Reviewed [14] |
|  | 60- to 70-nt | Reviewed [15] |
|  | ~70-nt | Experimental [16,17] & Summarized [4] |
|  | 70- to 90-nt | Summarized [18] |
|  | ~65-nt | Experimental [4] |
| Length of siRNA | 21- to 23-bp | Reviewed [19] & Experimental [20] |
|  | 20- to 23-bp | Reviewed [19] |
|  | 20- to 25-bp | Reviewed [1,19] |
|  | 21- to 22-bp | Experimental [21] & Experimental [10] |
|  | 24- to 26-bp | Experimental [21] |
|  | 35 or 22nt | Experimental [22] |
|  | ~25-nt | Experimental [23] |
|  | 21-nt | Experimental [24] |
|  |  |  |
| MiLength | 17- to 24-bp | Reviewed [19] |
|  | 20- to 29-bp | Reviewed [19] |
|  | 18- to 27-bp | Reviewed [19] |
|  | 20- to 24-bp | Reviewed [19] |
|  | 21- or 22-bp | Experimental [25] & Summarized [26] |
|  | 20- to 25-bp | Summarized [1] |
|  | ~22nt | Reviewed [1] & Experimental [3,22,27] & Summarized [28] |
|  | 18- to 24-nt | Experimental [10] |
|  | 21- to 25-nt | Reviewed [29-31] |
|  | 21- to 24-nt | Used [18] & Experimental [10,32] |
|  | ~25-nt | Experimental [33] |
|  | 18- to 23-nt | Experimental [34] |
|  | 19- to 27-nt | Experimental [35] |
|  | 17- to 27-nt | Experimental [36] |
|  | 25-bp, inactive | Experimental [12] |
| Stem | 45-nt | Experimental [37] |
| Basal Segment | Deleted, 10-bp, nonfunctional | Experimental [38] |
|  | Mutated, single strand, functional | Experimental [38] |
|  | Mutated, double paired, functional | Experimental [38] |
|  | Replaced, 10-bp or 12-bp, functional | Experimental [38] |
| Lower Stem | Shorted, 1-bp , functional | Experimental [39] |
|  | Shorted, 2-bp to 4-bp, decreased | Experimental [39] |
|  | Shorted, 5-bp, non-functional | Experimental [2] |
|  | Changed, 3-bp, functional | Experimental [40] |
|  | Changed & Enlarged, 6-bp or 10-bp, functional | Experimental [2] |
|  | Enlarged, 10-bp,nonfunctional | Experimental [2] |
| Upper Stem | Inserted, 2-bp or 4-bp, functional | Experimental [38,39] |
|  | Shorted, 1-bp or 2-bp/nt or 6-bp, functional | Experimental [38-40] |
|  | Shorted, 2-bp to 4-bp, decreased | Experimental [39] |
|  | Shorted, 5-bp to 8-bp, nonfunctional | Experimental [39] |
|  | Changed, 2-bp, functional | Experimental [40] |
|  | Changed, 1-nt, nonfunctional | Experimental [2] |
|  | ~20-bp and >18bp | Summarized [5] |
| Terminal Loop | Opened, 6-bp or 9-bp, functional | Experimental [38] |
|  | Diminished, 8-nt to 4-nt, functional | Experimental [2] |
|  | Diminished, 15-nt to 10-nt or 8-nt to 6-nt, decreased | Experimental [2,40] |
|  | Changed, 1-nt, 3-nt or 7-nt, functional | Experimental [2,40] |
|  | Changed & Diminished, 15-nt to >11-nt, functional | Experimental [2,40] |
|  | Changed & Diminished, 15-nt to <9-nt, nonfunctional | Experimental [2] |
|  | Enlarged, 8-nt to 10-nt, functional | Experimental [2] |
|  | Paired or Open the Terminal Loop and Circle the Basal Segment, cleavage site unchanged | Experimental [38] |
|  | opening the terminal loop into a basal segment, two cleavage site | Experimental [38] |
|  | 4-nt, low expression | Experimental [40] |
|  | Changed sequence or shorted to 11nt, functional | Experimental [40] |
|  | ~4-nt to 23-nt, no effect | Experimental [13] |
|  | Chemical element modified, slightly decreased | Experimental [41] |
| GC% of pre-miRNAs | Higher GC% (monocots to dicots) | Summarized [42] |
|  | Higher AU% (than GC%) | Summarized [5,43] |
|  | Similar (different species) | Summarized [5] |
|  | Similar (other RNAs) | Summarized [5] |
|  | Lower GC% (virus to plant or animal ) | Summarized [44] |
| GC% of siRNAs | No association between RNAi and Tm | Experimental [45] |
|  | 32% to 74% | Experimental [41] |
|  | Higher GC% (than genome) | Summarized [42] |
| GC% of miRNAs | Higher GC% (than pre-miRNAs) | Summarized [42] |
|  | Higher GC% (conserved to non-conserved) | Summarized [42] |
| Nucleotide content | No preference | Experimental [23] |
|  | No preference (Uracil) | Experimental [23] |
|  | Important (specificity) | Summarized [28] |
|  | Important (Dicer cleavage) | Summarized [28] |
| MFE | -0.4308±0.0025 (vertebrate) | Summarized [5] |
|  | -0.4456±0.0038 (plants) | Summarized [5] |
|  | Lower (than Random) | Summarized [46] |
|  | Lower (than tRNA or rRNA) | Summarized [46] |
|  | -35.8±8.7 kcal/mol (viral pre-miRNAs) | Summarized [44] |
| AMFE | MFEI2, -0.0761±0.0013 (vertebrate) | Summarized [5] |
|  | MFEI2, -0.0539±0.0010 (plan) | Summarized [5] |
|  | AMFE, -45.6 kcal/mol (virus) | Summarized [44] |
|  | AMFE, –45.93 ± 9.43 kcal/mol (plant) | Summarized [43] |
| MFEI | MFEI1, -0.0091±0.0001 (vertebrate) | Summarized [5] |
|  | MFEI1, -0.0096±0.0001 (plant) | Summarized [5] |
|  | Lower (pre-miRNAs in viral to plant) | Summarized [44] |
|  | ≧0.85 | Unknow [43] |
| Internal Loop of pre-miRNAs | Paired, 1-bp or 4-bp, functional | Experimental [37,39,40] |
|  | Created, 1- to 2-nt, functional | Experimental [39] |
|  | Created, 1- to 2-nt, decreased | Experimental [2,40] |
|  | Created, 3-nt, nonfunctional | Experimental [40] |
|  | Created, unknown, nonfunctional | Experimental [13] |
|  | Changed, 1-nt or 3-nt, functional | Experimental [40] |
|  | Enlarged, 2-nt, decreased | Experimental [39] |
|  | Enlarged, 3- to 4-nt, nonfunctional | Experimental [37,40] |
| Internal Loop of siRNAs | Positions 2-5, alter loading to RISC | Experimental [47] |
|  | Positions 6-15, no rules | Experimental [47] |
| Unpaired Rate | ~70.36–70.9% (base-pairing propensity) | Summarized [5] |
| GU Wobbles | Changed, 1-bp, functional | Experimental [2,40] |
|  | Created, 1-bp, functional | Experimental [40] |
|  | Induced, 1-bp, Increased asymmetry | Experimental [47] |
|  | Initial G:U wobble, directed the asymmetric incorporation to RISC | Experimental [47] |
| Strand | 5' strand | Experimental [48] |
|  | 5' strand | Experimental [47] |
|  | 3’ strand | Experimental [23] |
|  | Both | Reviewed [47] |
|  | Either | Experimental [32] |
|  | Both | Experimental [20] |
|  | Either | Experimental [23] |
| Stability | Weaker hydrogen bonding at its 5' end | Reviewed [2] |
|  | Low stability of the 5' end of the Antisense strand compared to the 5' end of the Sense strand | Experimental [47,49] |
|  | Decreased thermodynamic stability in the region of 10–14 (count from AS) | Experimental [49] |
|  | Stabilities determines which strand participates in the RNAi pathway | Reviewed [49] |
| 1^st^ Base of miRNAs | U | Summarized [32,44,50,51] |
|  | High U (Human) | Summarized [14] |
|  | Low U (C.elegans) | Summarized [14] |
|  | No G | Summarized [32] |
|  | non-sequence-specific recognition | Summarized [52] |
| 3’ overhang | ‘CC’ to ‘GG’, functional | Experimental [26] |
|  | Change to DNA, functional | Experimental [26] |
|  | ‘UG’, ‘UU’, ‘TT’, perfect | Experimental [26] |
|  | ‘UU’ | Surmmarized [53] |
|  | ‘UU’, ‘AG’ | Experimental [54] |
|  | 5’ overhang, functional | Experimental [12] |
|  | Overhang on antisense strand is more potent than sense strand | Experimental [26,28] |
| Overhang length | 1- to 4-nt, functional | Experimental [26] |
|  | 1- to 3-nt, functional | Experimental [28] |
|  | 2- to 3-nt, functional | Experimnetal [10] |
|  | >3-nt, Reduced | Experimental [28] |
|  | 17- to 20-nt, blocking | Experimental [26] |
|  | 0-nt (blunt), functional | Experimental [11,12,28] |
|  | 0-nt (blunt), nonfunctional | Summarized [53,55] |
| Penultimate Position | C > U = G > A | Experimental [28] |
| Terminal Nucleotide | A > G = U > C | Experimental [28] |

**References:**

1. Ambros, V.Bartel, B.Bartel, D. P.Burge, C. B.Carrington, J. C.et al. (2003) A uniform system for microRNA annotation. RNA 9: 277-279.

2. Zeng, Y.Yi, R.Cullen, B. R. (2005) Recognition and cleavage of primary microRNA precursors by the nuclear processing enzyme Drosha. EMBO J 24: 138-148.

3. Lee, R. C.Ambros, V. (2001) An extensive class of small RNAs in Caenorhabditis elegans. Science 294: 862-864.

4. Lee, Y.Jeon, K.Lee, J. T.Kim, S.Kim, V. N.et al. (2002) MicroRNA maturation: stepwise processing and subcellular localization. EMBO J 21: 4663-4670.

5. Ng, Kwang LoongMishra, S. K. (2007) Unique folding of precursor microRNAs: quantitative evidence and implications for de novo identification. RNA 13: 170-187.

6. Ngo, H.Tschudi, C.Gull, K.Ullu, E. (1998) Double-stranded RNA induces mRNA degradation in Trypanosoma brucei. Proc Natl Acad Sci U S A 95: 14687-14692.

7. Tuschl, T.Zamore, P. D.Lehmann, R.Bartel, D. P.Sharp, P. A.et al. (1999) Targeted mRNA degradation by double-stranded RNA in vitro. Genes Dev 13: 3191-3197.

8. Caplen, N. J.Fleenor, J.Fire, A.Morgan, R. A. (2000) dsRNA-mediated gene silencing in cultured Drosophila cells: a tissue culture model for the analysis of RNA interference. Gene 252: 95-105.

9. Hammond, S. M.Bernstein, E.Beach, D.Hannon, G. J. (2000) An RNA-directed nuclease mediates post-transcriptional gene silencing in Drosophila cells. Nature 404: 293-296.

10. Elbashir, S. M.Lendeckel, W.Tuschl, T. (2001) RNA interference is mediated by 21- and 22-nucleotide RNAs. Genes Dev 15: 188-200.

11. Zhang, H.Kolb, F. A.Brondani, V.Billy, E.Filipowicz, W.et al. (2002) Human Dicer preferentially cleaves dsRNAs at their termini without a requirement for ATP. EMBO J 21: 5875-5885.

12. Kim, D. H.Behlke, M. A.Rose, S. D.Chang, M. S.Choi, S.et al. (2005) Synthetic dsRNA Dicer substrates enhance RNAi potency and efficacy. Nat Biotechnol 23: 222-226.

13. Paddison, P. J.Caudy, A. A.Bernstein, E.Hannon, G. J.Conklin, D. S.et al. (2002) Short hairpin RNAs (shRNAs) induce sequence-specific silencing in mammalian cells. Genes Dev 16: 948-958.

14. Krol, J.Sobczak, K.Wilczynska, U.Drath, M.Jasinska, A.et al. (2004) Structural features of microRNA (miRNA) precursors and their relevance to miRNA biogenesis and small interfering RNA/short hairpin RNA design. J Biol Chem 279: 42230-42239.

15. Gregory, R. I.Yan, K. P.Amuthan, G.Chendrimada, T.Doratotaj, B.et al. (2004) The Microprocessor complex mediates the genesis of microRNAs. Nature 432: 235-240.

16. Pasquinelli, A. E.Reinhart, B. J.Slack, F.Martindale, M. Q.Kuroda, M. I.et al. (2000) Conservation of the sequence and temporal expression of let-7 heterochronic regulatory RNA. Nature 408: 86-89.

17. Lagos-Quintana, M.Rauhut, R.Lendeckel, W.Tuschl, T. (2001) Identification of novel genes coding for small expressed RNAs. Science 294: 853-858.

18. Grad, Y.Aach, J.Hayes, G. D.Reinhart, B. J.Church, G. M.et al. (2003) Computational and experimental identification of C. elegans microRNAs. Mol Cell 11: 1253-1263.

19. Krol, J.Krzyzosiak, W. J. (2004) Structural aspects of microRNA biogenesis. IUBMB Life 56: 95-100.

20. Zamore, P. D.Tuschl, T.Sharp, P. A.Bartel, D. P. (2000) RNAi: double-stranded RNA directs the ATP-dependent cleavage of mRNA at 21 to 23 nucleotide intervals. Cell 101: 25-33.

21. Hamilton, A.Voinnet, O.Chappell, L.Baulcombe, D. (2002) Two classes of short interfering RNA in RNA silencing. EMBO J 21: 4671-4679.

22. Bernstein, E.Caudy, A. A.Hammond, S. M.Hannon, G. J. (2001) Role for a bidentate ribonuclease in the initiation step of RNA interference. Nature 409: 363-366.

23. Parrish, S.Fleenor, J.Xu, S.Mello, C.Fire, A.et al. (2000) Functional anatomy of a dsRNA trigger: differential requirement for the two trigger strands in RNA interference. Mol Cell 6: 1077-1087.

24. Elbashir, S. M.Harborth, J.Lendeckel, W.Yalcin, A.Weber, K.et al. (2001) Duplexes of 21-nucleotide RNAs mediate RNA interference in cultured mammalian cells. Nature 411: 494-498.

25. Zhang, H.Kolb, F. A.Jaskiewicz, L.Westhof, E.Filipowicz, W.et al. (2004) Single processing center models for human Dicer and bacterial RNase III. Cell 118: 57-68.

26. Rose, S. D.Kim, D. H.Amarzguioui, M.Heidel, J. D.Collingwood, M. A.et al. (2005) Functional polarity is introduced by Dicer processing of short substrate RNAs. Nucleic Acids Res 33: 4140-4156.

27. Mourelatos, Z.Dostie, J.Paushkin, S.Sharma, A.Charroux, B.et al. (2002) miRNPs: a novel class of ribonucleoproteins containing numerous microRNAs. Genes Dev 16: 720-728.

28. Vermeulen, A.Behlen, L.Reynolds, A.Wolfson, A.Marshall, W. S.et al. (2005) The contributions of dsRNA structure to Dicer specificity and efficiency. RNA 11: 674-682.

29. Pham, J. W.Pellino, J. L.Lee, Y. S.Carthew, R. W.Sontheimer, E. J.et al. (2004) A Dicer-2-dependent 80s complex cleaves targeted mRNAs during RNAi in Drosophila. Cell 117: 83-94.

30. Parker, J. S.Roe, S. M.Barford, D. (2004) Crystal structure of a PIWI protein suggests mechanisms for siRNA recognition and slicer activity. EMBO J 23: 4727-4737.

31. Hammond, S. M.Boettcher, S.Caudy, A. A.Kobayashi, R.Hannon, G. J.et al. (2001) Argonaute2, a link between genetic and biochemical analyses of RNAi. Science 293: 1146-1150.

32. Lau, N. C.Lim, L. P.Weinstein, E. G.Bartel, D. P. (2001) An abundant class of tiny RNAs with probable regulatory roles in Caenorhabditis elegans. Science 294: 858-862.

33. Hamilton, A. J.Baulcombe, D. C. (1999) A species of small antisense RNA in posttranscriptional gene silencing in plants. Science 286: 950-952.

34. Lagos-Quintana, M.Rauhut, R.Meyer, J.Borkhardt, A.Tuschl, T.et al. (2003) New microRNAs from mouse and human. RNA 9: 175-179.

35. Lim, L. P.Lau, N. C.Weinstein, E. G.Abdelhakim, A.Yekta, S.et al. (2003) The microRNAs of Caenorhabditis elegans. Genes Dev 17: 991-1008.

36. Aravin, A. A.Lagos-Quintana, M.Yalcin, A.Zavolan, M.Marks, D.et al. (2003) The small RNA profile during Drosophila melanogaster development. Dev Cell 5: 337-350.

37. Lee, Y.Ahn, C.Han, J.Choi, H.Kim, J.et al. (2003) The nuclear RNase III Drosha initiates microRNA processing. Nature 425: 415-419.

38. Han, J.Lee, Y.Yeom, K. H.Nam, J. W.Heo, I.et al. (2006) Molecular Basis for the Recognition of Primary microRNAs by the Drosha-DGCR8 Complex. Cell 125: 887-901.

39. Chelladurai, B.Li, H.Zhang, K.Nicholson, A. W. (1993) Mutational analysis of a ribonuclease III processing signal. Biochemistry 32: 7549-7558.

40. Zeng, Y.Cullen, B. R. (2003) Sequence requirements for micro RNA processing and function in human cells. RNA 9: 112-123.

41. Harborth, J.Elbashir, S. M.Vandenburgh, K.Manninga, H.Scaringe, S. A.et al. (2003) Sequence, chemical, and structural variation of small interfering RNAs and short hairpin RNAs and the effect on mammalian gene silencing. Antisense Nucleic Acid Drug Dev 13: 83-105.

42. Ho, T.Wang, H.Pallett, D.Dalmay, T. (2007) Evidence for targeting common siRNA hotspots and GC preference by plant Dicer-like proteins. FEBS Lett 581: 3267-3272.

43. Zhang, B. H.Pan, X. P.Cox, S. B.Cobb, G. P.Anderson, T. A.et al. (2006) Evidence that miRNAs are different from other RNAs. Cell Mol Life Sci 63: 246-254.

44. Pan, X.Zhang, B.Francisco, M. S.Cobb, G. P. (2006) Characterizing viral microRNAs and its application on identifying new microRNAs in viruses. J Cell Physiol

45. Hohjoh, H. (2002) RNA interference (RNA(i)) induction with various types of synthetic oligonucleotide duplexes in cultured human cells. FEBS Lett 521: 195-199.

46. Bonnet, E.Wuyts, J.Rouze, P.Van, Peer Y. (2004) Evidence that microRNA precursors, unlike other non-coding RNAs, have lower folding free energies than random sequences. Bioinformatics 20: 2911-2917.

47. Schwarz, D. S.Hutvagner, G.Du, T.Xu, Z.Aronin, N.et al. (2003) Asymmetry in the assembly of the RNAi enzyme complex. Cell 115: 199-208.

48. Lin, S. L.Chang, D.Ying, S. Y. (2005) Asymmetry of intronic pre-miRNA structures in functional RISC assembly. Gene 356: 32-38.

49. Khvorova, A.Reynolds, A.Jayasena, S. D. (2003) Functional siRNAs and miRNAs exhibit strand bias. Cell 115: 209-216.

50. Zhang, B.Pan, X.Cannon, C. H.Cobb, G. P.Anderson, T. A.et al. (2006) Conservation and divergence of plant microRNA genes. Plant J 46: 243-259.

51. Bartel, D. P. (2004) MicroRNAs: genomics, biogenesis, mechanism, and function. Cell 116: 281-297.

52. Ma, J. B.Yuan, Y. R.Meister, G.Pei, Y.Tuschl, T.et al. (2005) Structural basis for 5'-end-specific recognition of guide RNA by the A. fulgidus Piwi protein. Nature 434: 666-670.

53. Song, J. J.Liu, J.Tolia, N. H.Schneiderman, J.Smith, S. K.et al. (2003) The crystal structure of the Argonaute2 PAZ domain reveals an RNA binding motif in RNAi effector complexes. Nat Struct Biol 10: 1026-1032.

54. Ma, J. B.Ye, K.Patel, D. J. (2004) Structural basis for overhang-specific small interfering RNA recognition by the PAZ domain. Nature 429: 318-322.

55. Lingel, A.Simon, B.Izaurralde, E.Sattler, M. (2003) Structure and nucleic-acid binding of the Drosophila Argonaute 2 PAZ domain. Nature 426: 465-469.

## Supplementary Table 2: Summary of mi-RNA or pre-miRNA prediction software tools.

| Software | Links | Year | Method | Function | Available | Type | Ref |
| --- | --- | --- | --- | --- | --- | --- | --- |
| MiRscan | http://genes.mit.edu/cgi-bin/mirscan.pl | 2003 | Homologous Search | miRNA | NO | WEB | [1] |
| miRseeker | not found | 2003 | Conserve Searching | miRNA | N.A | N.A | [2] |
| miRCheck | not found | 2004 | Conserve Searching | miRNA | N.A | N.A | [3] |
| findMiRNA | not found | 2005 | miRNA:target pairs | miRNA | N.A | N.A | [4] |
| *miRAlign | http://bioinfo.au.tsinghua.edu.cn/miralign/ | 2005 | Sequence and Structure Alignment | miRNA | YES | WEB | [5] |
| *ab initio classifier* | http://bioinfo.au.tsinghua.edu.cn/mirnasvm | 2005 | Support Vector Machine | pre-miRNA | YES | LOCAL | [6] |
| PalGrade | not found | 2005 | Conserve Searching | miRNA | N.A | N.A | [7] |
| miR-abela | http://www.mirz.unibas.ch/cgi/pred_miRNA_genes.cgi | 2005 | Cross-species searching | pre-miRNA | YES | WEB | [8] |
| *BayesMiRNAfind | https://bioinfo.wistar.upenn.edu/miRNA/miRNA/ | 2006 | Naïve Bayes classifier | miRNA | YES | WEB | [9] |
| RNAmicro | http://www.bioinf.uni-leipzig/Software/RNAmicro | 2006 | Support Vector Machine | pre-miRNA | NO | LOCAL | [10] |
| Cons-SVM | not found | 2007 | Support Vector Machine | miRNA | N.A | N.A | [11] |
| Microprocessor SVM | https://demo1.interagon.com/miRNA/ | 2007 | Support Vector Machine | miRNA | NO | BOTH | [12] |
| miRPred | not found | 2007 | Linear Genetic Programming | miRNA | NO | N.A | [13] |
| MiRFinder | http://www.bioinformatics.org/mirfinder/ | 2007 | Support Vector Machine | pre-miRNA | YES | LOCAL | [14] |
| miPred | http://web.bii.a-star.edu.sg/~stanley/Publications | 2007 | Support Vector Machine | pre-miRNA | NO | WEB | [15] |
| MiPred | http://www.bioinf.seu.edu.cn/miRNA/ | 2007 | Random Forest (RF) | pre-miRNA | YES | WEB | [16] |
| miRNAFinder | http://bioinfo3.noble.org/mirna/ | 2007 | Support Vector Machine | miRNA | YES | WEB | [14] |
| *mirEval | http://tagc.univ-mrs.fr/mireval | 2008 | Triplet-SVM | miRNA | YES | WEB | [17] |
| miRNAminer | http://pag.csail.mit.edu/mirnaminer | 2008 | Homologous Search | N.A | N.A | WEB | [18] |
| No name | not found | 2008 | Support Vector Machine | pre-miRNA | NO | N.A | [19] |
| miR-KDE | not found | 2008 | Relaxed Variable Kernel Density Estimator (RVKDE) | pre-miRNA | NO | N.A | [20] |
| miRank | Matlab | 2008 | Random Walks | miRNA | YES | LOCAL | [21] |

* indicates software that was selected for comparsion with our software package.

Software marked with asterisk was selected for comparison with miRPara. Software was selected according to whether (i) it could predict miRNA, (ii) could analyze test data comprising of multiple sequences in a reasonable time, and (iii) whether the software or website was still available.

**References**

1. Lim LP, Lau NC, Weinstein EG, et al.: **The microRNAs of Caenorhabditis elegans**. *Genes & Development* 2003, **17**:991-1008.

2. Lai EC, Tomancak P, Williams RW, Rubin GM: **Computational identification of Drosophila microRNA genes**. *Genome Biology* 2003, **4**:R42.

3. Jones-Rhoades MW, Bartel DP: **Computational identification of plant microRNAs and their targets, including a stress-induced miRNA**. *Molecular Cell* 2004, **14**:787-99.

4. Adai A, Johnson C, Mlotshwa S, et al.: **Computational prediction of miRNAs in Arabidopsis thaliana**. *Genome Research* 2005, **15**:78-91.

5. Wang X, Zhang J, Li F, et al.: **MicroRNA identification based on sequence and structure alignment**. *Bioinformatics (Oxford, England)* 2005, **21**:3610-4.

6. Xue C, Li F, He T, et al.: **Classification of real and pseudo microRNA precursors using local structure-sequence features and support vector machine**. *BMC Bioinformatics* 2005, **6**:310.

7. Bentwich I, Avniel A, Karov Y, et al.: **Identification of hundreds of conserved and nonconserved human microRNAs**. *Nature Genetics* 2005, **37**:766-70.

8. Sewer A, Paul N, Landgraf P, et al.: **Identification of clustered microRNAs using an ab initio prediction method**. *BMC Bioinformatics* 2005, **6**:267.

9. Yousef M, Nebozhyn M, Shatkay H, et al.: **Combining multi-species genomic data for microRNA identification using a Naive Bayes classifier**. *Bioinformatics (Oxford, England)* 2006, **22**:1325-34.

10. Hertel J, Stadler PF: **Hairpins in a Haystack: recognizing microRNA precursors in comparative genomics data**. *Bioinformatics (Oxford, England)* 2006, **22**:e197-202.

11. Gu J, Fu H, Zhang X, Li Y: **Identifications of conserved 7-mers in 3'-UTRs and microRNAs in Drosophila**. *BMC Bioinformatics* 2007, **8**:432.

12. Helvik SA, Snøve O, Saetrom P: **Reliable prediction of Drosha processing sites improves microRNA gene prediction**. *Bioinformatics (Oxford, England)* 2007, **23**:142-9.

13. Brameier M, Wiuf C: **Ab initio identification of human microRNAs based on structure motifs**. *BMC Bioinformatics* 2007, **8**:478.

14. Huang T, Fan B, Rothschild MF, et al.: **MiRFinder: an improved approach and software implementation for genome-wide fast microRNA precursor scans**. *BMC Bioinformatics* 2007, **8**:341.

15. Ng KL, Mishra SK: **De novo SVM classification of precursor microRNAs from genomic pseudo hairpins using global and intrinsic folding measures**. *Bioinformatics (Oxford, England)* 2007, **23**:1321-30.

16. Jiang P, Wu H, Wang W, et al.: **MiPred: classification of real and pseudo microRNA precursors using random forest prediction model with combined features**. *Nucleic Acids Research* 2007, **35**:W339-44.

17. Ritchie W, Théodule F, Gautheret D: **Mireval: a web tool for simple microRNA prediction in genome sequences**. *Bioinformatics (Oxford, England)* 2008, **24**:1394-6.

18. Artzi S, Kiezun A, Shomron N: **miRNAminer: a tool for homologous microRNA gene search**. *BMC Bioinformatics* 2008, **9**:39.

19. Xu J, Li F, Sun Q: **Identification of microRNA precursors with support vector machine and string kernel**. *Genomics, Proteomics & Bioinformatics / Beijing Genomics Institute* 2008, **6**:121-8.

20. Chang DT, Wang C, Chen J: **Using a kernel density estimation based classifier to predict species-specific microRNA precursors**. *BMC Bioinformatics* 2008, **9 Suppl 12**:S2.

21. Xu Y, Zhou X, Zhang W: **MicroRNA prediction with a novel ranking algorithm based on random walks**. *Bioinformatics (Oxford, England)* 2008, **24**:i50-8.

## Supplementary Table 3: Additional Results from comparison of miRPara with other miRNA prediction software

| **miRBase ID** | **Species** |
| --- | --- |
| bta-mir-2487 | Metazoa |
| dre-mir-222b | Metazoa |
| sme-mir-2148 | Metazoa |
| hsa-mir-764 | Metazoa |
| bta-mir-2375 | Metazoa |
| bmo-mir-2740 | Metazoa |
| sme-mir-61b | Metazoa |
| sme-mir-2169 | Metazoa |
| bta-mir-2481 | Metazoa |
| mtr-MIR2119 | Viridiplantae |
| bta-mir-2408 | Metazoa |
| bta-mir-2322 | Metazoa |
| bta-mir-2305 | Metazoa |
| bta-mir-2350 | Metazoa |
| bta-mir-2470 | Metazoa |
| bta-mir-2411 | Metazoa |
| dre-mir-2197 | Metazoa |
| pvu-MIR482 | Viridiplantae |
| hvt-mir-H13 | Viruses |
| bta-mir-2435 | Metazoa |
| pvu-MIR166a | Viridiplantae |
| cel-mir-2215 | Metazoa |
| bta-mir-2485 | Metazoa |
| bta-mir-2284s | Metazoa |
| bmo-mir-2738 | Metazoa |
| bta-mir-2334 | Metazoa |
| bta-mir-2361 | Metazoa |
| mtr-MIR172 | Viridiplantae |
| bmo-mir-2733a | Metazoa |
| bta-mir-2297 | Metazoa |
| sme-mir-2156b | Metazoa |
| bta-mir-2468 | Metazoa |
| dre-mir-107b | Metazoa |
| gma-MIR167e | Viridiplantae |
| mtr-MIR390 | Viridiplantae |
| mmu-mir-2143-1 | Metazoa |
| bta-mir-2330 | Metazoa |
| bta-mir-2341 | Metazoa |
| bta-mir-339b | Metazoa |
| bta-mir-2449 | Metazoa |
| sme-mir-216 | Metazoa |
| osa-MIR2118i | Viridiplantae |
| bta-mir-2345 | Metazoa |
| peu-MIR2912a | Viridiplantae |
| bta-mir-2307 | Metazoa |
| sme-mir-2f | Metazoa |
| hvt-mir-H4 | Viruses |
| bta-mir-2392 | Metazoa |
| mtr-MIR167 | Viridiplantae |
| bta-mir-2313 | Metazoa |
| bta-mir-2348 | Metazoa |
| bmo-mir-2744 | Metazoa |
| bta-mir-2318 | Metazoa |
| bta-mir-2325a | Metazoa |
| peu-MIR2913 | Viridiplantae |
| mmu-mir-2141 | Metazoa |
| osa-MIR2124c | Viridiplantae |
| mmu-mir-2134-2 | Metazoa |
| sme-mir-754b-2 | Metazoa |
| bta-mir-2397 | Metazoa |
| bta-mir-2329-1 | Metazoa |
| hsa-mir-548q | Metazoa |
| hsa-mir-2278 | Metazoa |
| dre-mir-196c | Metazoa |
| hvt-mir-H18 | Viruses |
| sme-mir-2147d | Metazoa |
| bta-mir-2310 | Metazoa |
| bta-mir-2454 | Metazoa |
| mmu-mir-2134-3 | Metazoa |
| bta-mir-2455 | Metazoa |
| hsa-mir-2114 | Metazoa |
| bta-mir-2405 | Metazoa |
| bta-mir-320b | Metazoa |
| bta-mir-2389 | Metazoa |
| osa-MIR2124b | Viridiplantae |
| bta-mir-2424-1 | Metazoa |
| sme-mir-2205 | Metazoa |
| bmo-mir-2747 | Metazoa |
| sme-mir-2204 | Metazoa |
| bmo-mir-2731b | Metazoa |
| sme-mir-2147c | Metazoa |
| pvu-MIR2118 | Viridiplantae |
| sme-mir-87c | Metazoa |
| bta-mir-2378 | Metazoa |
| bmo-mir-2753 | Metazoa |
| mtr-MIR396a | Viridiplantae |
| bta-mir-2338 | Metazoa |
| bta-mir-2474 | Metazoa |
| bta-mir-1721 | Metazoa |
| bta-mir-2355 | Metazoa |
| mmu-mir-2132 | Metazoa |
| bmo-mir-2726 | Metazoa |
| dre-mir-2194 | Metazoa |
| bta-mir-2384-1 | Metazoa |
| bta-mir-2284i | Metazoa |
| bta-mir-1777a | Metazoa |
| mtr-MIR2199 | Viridiplantae |
| sme-mir-7d | Metazoa |
| bmo-mir-2732 | Metazoa |
| bta-mir-2398 | Metazoa |

List of miRNAs that predicted from the NEW dataset in the software comparison test that were not found in miRBase but which were subsequently verified in a later release
